# The molecular and gene/miRNA expression profiles of radioiodine resistant papillary thyroid cancer

**DOI:** 10.1186/s13046-020-01757-x

**Published:** 2020-11-16

**Authors:** Carla Colombo, Emanuela Minna, Chiara Gargiuli, Marina Muzza, Matteo Dugo, Loris De Cecco, Gabriele Pogliaghi, Delfina Tosi, Gaetano Bulfamante, Angela Greco, Laura Fugazzola, Maria Grazia Borrello

**Affiliations:** 1 Department of Pathophysiology and Transplantation, Università degli Studi di Milano, Milan, Italy; 2 Division of Endocrine and Metabolic Diseases, IRCCS Istituto Auxologico Italiano, Milan, Italy; 3 Department of Research, Molecular Mechanisms Unit, Fondazione IRCCS Istituto Nazionale dei Tumori, Milan, Italy; 4 Department of Applied Research and Technology Development, Platform of Integrated Biology, Fondazione IRCCS Istituto Nazionale dei Tumori, Milan, Italy; 5 Laboratory of Endocrine and Metabolic Research, IRCCS Istituto Auxologico Italiano, Milan, Italy; 6 Department of Health Sciences, Division of Human Pathology, Università degli Studi di Milano, Milan, Italy

**Keywords:** Thyroid, Oncogenes, Radioiodine refractory, Gene/miRNA profiles, Papillary thyroid cancer

## Abstract

**Background:**

Papillary thyroid cancer (PTC) is the most frequent endocrine tumor. Radioiodine (RAI) treatment is highly effective in these tumors, but up to 60% of metastatic cases become RAI-refractory. Scanty data are available on either the molecular pattern of radioiodine refractory papillary thyroid cancers (PTC) or the mechanisms responsible for RAI resistance.

**Methods:**

We analyzed the molecular profile and gene/miRNA expression in primary PTCs, synchronous and RAI-refractory lymph node metastases (LNMs) in correlation to RAI avidity or refractoriness.

We classified patients as RAI+/D+ (RAI uptake/disease persistence), RAI−/D+ (absent RAI uptake/disease persistence), and RAI+/D- (RAI uptake/disease remission), and analyzed the molecular and gene/miRNA profiles, and the expression of thyroid differentiation (TD) related genes.

**Results:**

A different molecular profile according to the RAI class was observed: *BRAF*^*V600E*^ cases were more frequent in RAI−/D+ (*P* = 0.032), and fusion genes in RAI+/D+ cases. RAI+/D- patients were less frequently *pTERT* mutations positive, and more frequently wild type for the tested mutations/fusions. Expression profiles clearly distinguished PTC from normal thyroid. On the other hand, in refractory cases (RAI+/D+ and RAI−/D+) no distinctive PTC expression patterns were associated with either tissue type, or RAI uptake, but with the driving lesion and BRAF−/RAS-like subtype. Primary tumors and RAI-refractory LNMs with *BRAF*^*V600E*^ mutation display transcriptome similarity suggesting that RAI minimally affects the expression profiles of RAI-refractory metastases. Molecular profiles associated with the expression of *TPO*, *SLC26A4* and TD genes, that were found more downregulated in *BRAF*^*V600E*^ than in gene fusions tumors.

**Conclusions:**

The present data indicate a different molecular profile in RAI-avid and RAI-refractory metastatic PTCs. Moreover, *BRAF*^*V600E*^ tumors displayed reduced differentiation and intrinsic RAI refractoriness, while PTCs with fusion oncogenes are RAI-avid but persistent, suggesting different oncogene-driven mechanisms leading to RAI refractoriness.

**Supplementary Information:**

The online version contains supplementary material available at 10.1186/s13046-020-01757-x.

## Background

Radioactive iodine (RAI) therapy is a very effective treatment, significantly increasing the life expectancy of differentiated thyroid cancer (DTC) patients [1]. However, about 60% of metastatic tumors (corresponding to less than 5% of all thyroid cancers) will become radioiodine-refractory (RAI-R), with a major impact on patient prognosis. In particular, the 10 years survival rate significantly decreases in patients without any ^131^I uptake at diagnosis compared to patients with initial ^131^I uptake but persistent diseases, and even more if compared to RAI-avid (RAI-A) metastatic thyroid tumors [2].

According to American Thyroid Association (ATA) Guidelines [3], RAI-refractory DTCs are identified by: a) presence of malignant/metastatic tissue that does not ever concentrate RAI, b) presence of tumor tissue that loses the ability to concentrate RAI after previous evidence of RAI-avid disease, c) RAI uptake in some lesions but not in others, and d) metastatic disease that progresses despite significant concentration of RAI.

It was shown that *BRAF*^*V600E*^ mutated PTCs display a greater reduction in the expression of genes involved in iodine uptake and organification, namely sodium/iodide symporter *NIS* (*SLC5A5*), thyroperoxidase (*TPO*) and pendrin (*SLC26A4*), compared with tumors with other mutations or without known genetic alterations [4–6]. In vitro studies showed that the activation of *BRAF*^*V600E*^ down-regulates the expression of NIS and reduces RAI uptake in thyroid cells. On the other hand, no difference either in *NIS* or in other thyroid differentiation genes was found in PTC with or without *RET/PTC* rearrangements [7], in accordance with the notion that these tumors rarely progress to aggressive or undifferentiated carcinomas [8].

Few studies are currently available on the molecular profile of RAI-R thyroid cancers, either well differentiated, poorly differentiated or anaplastic, showing that they are enriched with *BRAF* mutations, whereas *RAS* mutations are more represented in RAI-A metastatic DTCs [9, 10]. Based on these in vitro and in vivo data, pilot studies and phase 2 trials demonstrated that two selective BRAF inhibitors, namely Vemurafenib and Dabrafenib, are able to stimulate radioiodine uptake in patients with metastatic *BRAF*^*V600E*^-mutant RAI-R DTCs [11–16]. In addition, attempts of reverse RAI refractoriness in TC patients restoring radioiodine uptake, have been conducted with MEK inhibitors, tested as single agent [17] or, more recently, in combination with BRAF inhibitors [18, 19].

Even though the aberrant expression and/or function of NIS has been identified as factor for the lack of sensitivity to RAI, the mechanisms responsible for RAI resistance are still poorly understood. To get more insights into this topic, we investigated both the molecular profiles of RAI-A and RAI-R PTC patients, and their gene/miRNA expression. Indeed, though some studies have already described miRNA deregulation in PTC suggesting their possible role as diagnostic and prognostic markers [20–24], any report is currently available about miRNA expression in relation to the response to RAI.

Therefore, aim of this study was to find possible molecular tags able to identify the avidity or refractoriness to radioiodine of PTCs, since this information is expected to have a major impact on the selection of the more appropriate treatment and follow up [25].

## Methods

### Patients

**Fig. 1:**
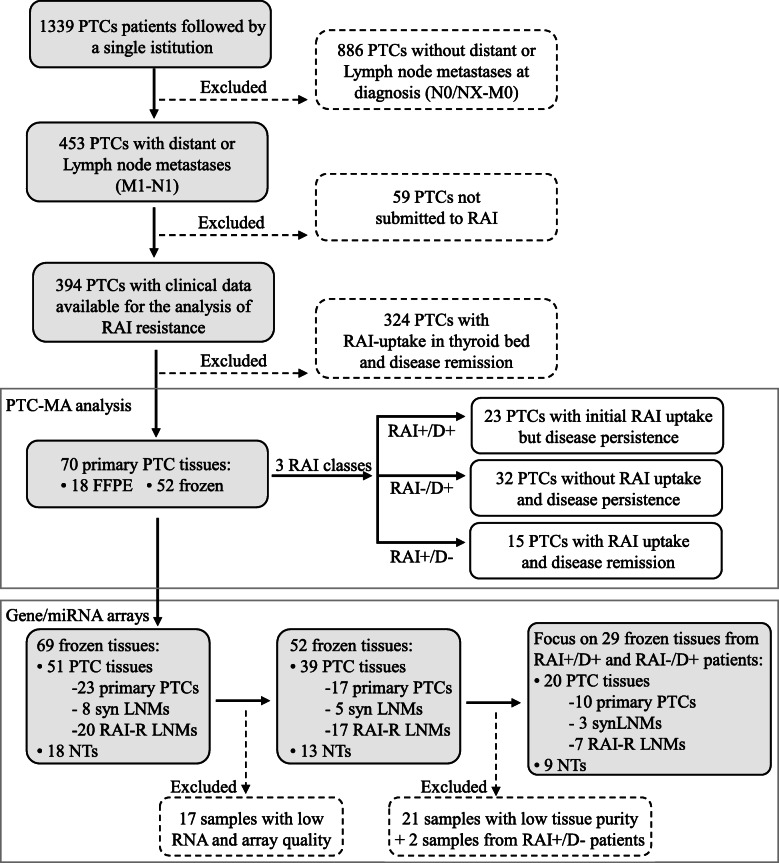
Flowchart of patients and tissues selection. Starting from a database of 1339 patients, subsequent strict selections were done in order to identify patients for whom a radioiodine (RAI) avidity or refractoriness could be definitely determined. Seventy primary papillary thyroid cancer (PTC) tissues were included, divided according to the RAI class (RAI+/D+: initial RAI uptake but disease persistence, RAI−/D+: without RAI uptake and disease persistence, and RAI+/D-: RAI uptake and disease remission), and submitted to the analyses of multiple PTC-related gene mutations and fusions by a PTC-Mass Array platform. The subset of tissues submitted to gene/miRNA arrays are reported. They included primary PTCs, synchronous lymph node metastasis (LNM), RAI refractory LNM (collected after RAI treatment), and non-neoplastic thyroid (NT)

We retrospectively analyzed, the clinical records of 1339 PTC patients treated and followed by our tertiary care center during the period 2001–2019 (Fig. 1). Strict selection criteria were applied to identify patients for whom RAI avidity or refractoriness could be definitely determined: first: patients without regional or distant metastases at diagnosis were excluded, due to the impossibility to determine RAI avidity or refractoriness (1339–886 = 453); second: patients with regional or distant metastases at diagnosis but not submitted to RAI treatment for several reasons (patient choice in most cases) were excluded (453–59 = 394); third: patients with RAI uptake limited to the thyroid bed at the time of ablation with following complete remission were excluded (394–324 = 70).

Finally, the selected 70 PTC patients, all with regional or distant metastases at diagnosis, were classified into three different groups according to RAI uptake and response: RAI+/D+: 23 PTCs with initial RAI uptake at the metastatic site, and disease persistence; RAI−/D+: 32 PTCs without RAI uptake at the metastatic site and disease persistence; and RAI+/D-: 15 PTCs with RAI uptake at the metastatic site and disease remission. In RAI+ groups, all metastases, either loco regional or distant, were RAI avid.

Patients were treated, followed up, and defined as in remission or in persistence according to the International and National guidelines [3, 26]. Patients were risk-stratified using AJCC staging system (Stage I, II,III or IV) [27] and 2015 ATA Guidelines (low, intermediate or high risk of recurrence) [3].

### Molecular profile by PTC-MA assay

Seventy primary PTCs (52 frozen and 18 FFPE tissues) and 22 lymph node metastases (frozen tissues) were submitted to molecular analyses by PTC-Mass Array platform (PTC-MA), previously set up for the simultaneous identification of 13 known hotspot mutations (*BRAF*^*V600E*^; *AKT1*^*E17K*^; *EIF1AX* c.338-1G > C; *NRAS*^*Q61R*^ and *NRAS*^*Q61K*^; *HRAS*^*G13C*^, *HRAS*^*Q61K*^, and *HRAS*^*Q61R*^; *KRAS*^*G12V*^ and *KRAS*^*G13C*^; *TERT* c.-124C > T and *TERT* c.-146C > T; and *PIK3CA*^*E542K*^) and 6 recurrent fusion genes typical of PTC: *RET/PTC1* (*RET/CCDC6*), *RET/PTC2* (*RET/PRKAR1A*), and *RET/PTC3* (*RET/NCOA4*) and *TRK* (*NTRK1/TPM3*), *TRK-T1* (*NTRK-T1/TPR*), and *TRK-T3* (*NTRK1/TFG*) [28, 29].

For point mutations the allelic frequencies were recorded and normalized for the cancer cell content, as previously described [29]. For fusion genes, the allelic frequency cannot be evaluated, since their detection involves a selective amplification of the rearranged gene transcript.

### Samples selection for gene/miRNA profiling

For 33/70 patients submitted to PTC-MA analysis, frozen samples of the primary tumor and/or the metastasis were still available after the genetic characterization, and were submitted to gene/miRNA microarray analysis. Whenever available, synchronous lymph node metastasis (synLNM) and/or RAI-R LNM diagnosed and collected after RAI treatment (LNM post RAI), and non-neoplastic thyroid (NT) were also studied. In order to increase the number of metastatic samples, 9 additional patients (for a total of 42 patients) were included, for whom only metastatic tumor tissue was available. Overall, 69 frozen tissues derived from those 42 PTC metastatic patients were collected including 23 primary tumors, 8 synLNMs, 20 LNMs post RAI, and 18 matched NTs (Fig. 1).

### Gene/miRNA microarray analysis

Nucleic acid extraction was performed on the 69 collected frozen tissues. Genomic DNA and RNA were extracted by Qiagen DNeasy Blood & Tissue Kit and by Qiagen miRNeasy Mini Kit, respectively, and quantified by Qubit 2.0 Fluorometer (Thermo Fisher). DNA and RNA quality and integrity were assessed using Agilent 2100 Bioanalyzer (Agilent Technologies, Palo Alto, CA, USA).

Gene profiles were established by Thermo Fisher Human Clariom S Assay. MiRNA profiles were established by Agilent SurePrint Human miRNA microarrays. Complete microarrays experimental procedures and data processing available in Additional file 2: Supplemental Methods.

Following quality control and data pre-processing, gene expression data were available for 52 thyroid tissues including 39 primary and LN metastatic PTCs and 13 NTs (Fig. 1).

The BRAF−/RAS-like subtype, indicative of MAPK pathway transcriptional activation, was assigned using the 71 gene signature derived from The Cancer Genome Atlas (TCGA) study on PTC [30] as previously described [31]; BRAF−/RAS-like subtype were further confirmed using the Nearest Template Prediction (NTP) algorithm [32] implemented in GenePattern software (https://www.genepattern.org/).

Unsupervised analyses for gene and miRNA arrays were performed using principal component analysis (PCA) and hierarchical clustering with Euclidean distance and Ward linkage method. Heatmaps were visualized using the ComplexHeatmap package [33]. Differential expression analysis was carried out using the limma package [34]. *P*-values were corrected for multiple testing using the Benjamini-Hochberg false discovery rate method.

CIBERSORT algorithm [35] was applied to compute tumor purity; *p*-value outputs were used to stratify samples (*p*-value < 0.05 were considered significantly infiltrated and assigned to a low tumor purity class, while *p*-value ≥0.05 were assigned to a high tumor purity class). According to CIBERSORT tumor purity, 21 samples, resulting with low purity, were excluded from the subsequent analysis (Fig. 1).

### Gene expression validation in public datasets

Human thyroid cancer gene datasets available on public repositories were investigated for genes expression validation. TCGA study on PTC [30] and nine additional datasets from GEO were analyzed (GSE27155 [36], GSE3467 [37], GSE6004 [38], GSE33630 [39, 40], GSE35570 [41], GSE53157 [42], GSE29265, GSE3678, and GSE60542 [43]). Details available in Additional file 2: Supplemental Methods. Both normal thyroid and primary PTC samples were tested. Three thyroid specific genes (*NIS/SLC5A5*, *TPO*, and *SLC24A6*) and a thyroid differentiation (TD) score were assessed; only PTCs with reported *BRAF*^*V600E*^ mutation or *RET* and *NTRK1* gene fusions were specifically studied. TD score was calculated as mean of log2-transformed and median-centered expression of 16 thyroid function related genes as previously described in TCGA study [30]; list of the 16 TD genes available in Additional file 1: Suppl. Figure S6E.

### Statistical analysis

Relations between discrete variables were evaluated by means of χ2 test or t-test, as appropriate. Clinicopathological and molecular features were evaluated by univariate analysis.

Statistical significance was defined as *P* < 0.05. All statistical analyses were performed using MedCalc Analyses using the Version 18.11.3 of the MedCalc Software (B-8400 Ostend, Belgium).

## Results

### Clinicopathological features

**Fig. 2:**
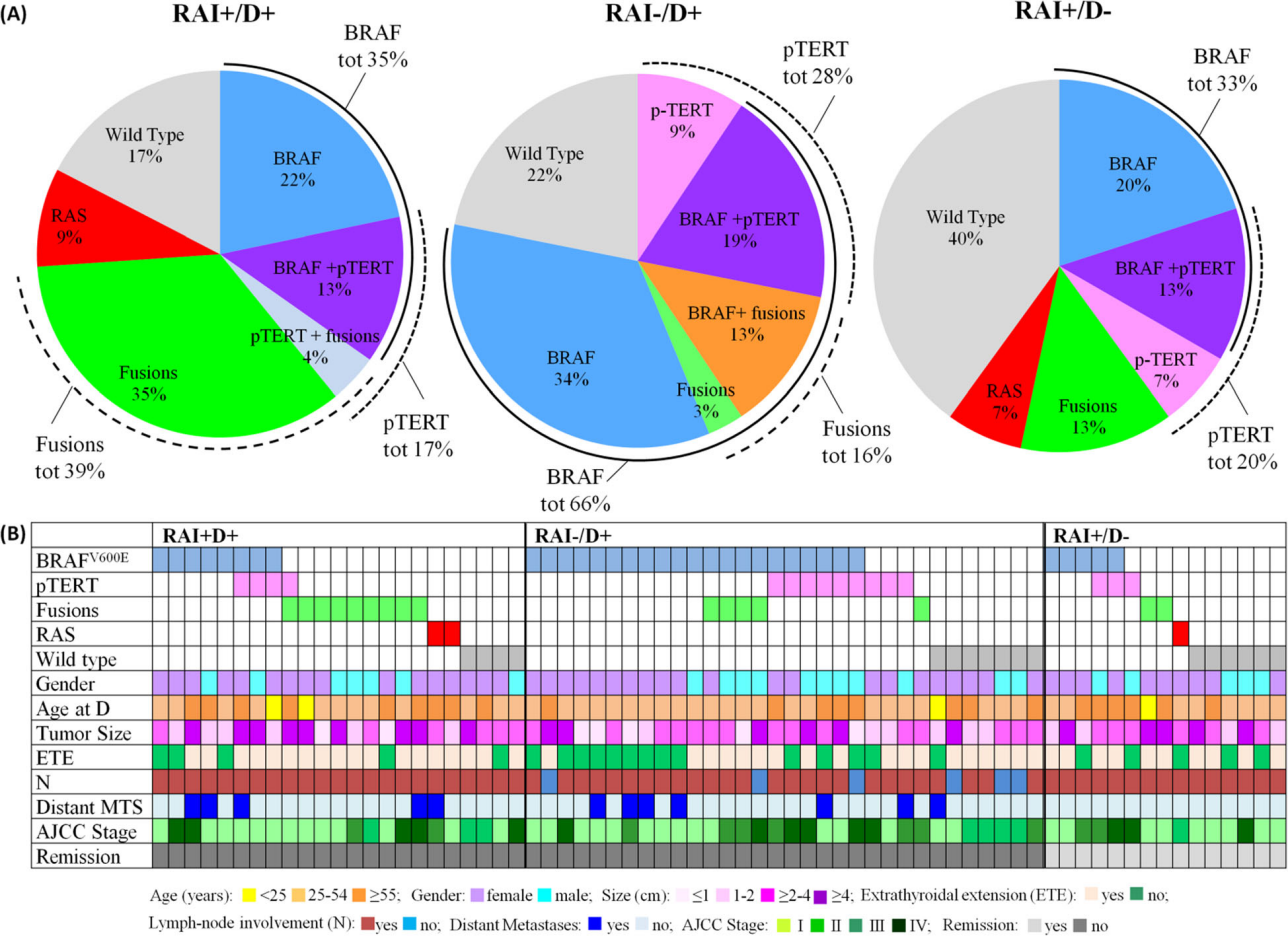
Molecular profiling by PTC-MA in 70 RAI-refractory and RAI-avid primary PTCs. **a** Percentage of cases harboring the different genetic alterations tested for each radioiodine (RAI) class (RAI+/D+, initial RAI uptake but disease persistence; RAI−/D+, without RAI uptake and disease persistence; and RAI+/D-, RAI uptake and disease remission). *BRAF*^*V600E*^ was statistically significant more prevalent in RAI−/D+ compared with the other 2 classes (66% vs 35 and 33%, *P* = 0.032). **b** Clinical and molecular features of the patients included in the 3 different RAI classes. Note that some patients had the coexistence of 2 different mutations. Abbreviations: N, Lymph-Node involvement; ETE, Extrathyroidal Extension; MTS, Metastases; AJCC, American Joint Committee on Cancer

The three groups of patients (RAI+/D+, RAI−/D+, and RAI+/D-) displayed comparable clinicopathological features at diagnosis, with minor differences observed only for the prevalence of the non-conventional histological variants of PTC. There were no differences regarding the most important prognostic criteria of differentiated thyroid cancer: TNM, AJCC stage, ATA risk classes (Fig. 2). The median cumulative dose of radioiodine was similar in the three groups (Table 1).

**Table 1:** Clinicopathologic features of the 70 patients classified according to radioiodine (RAI) avidity/refractoriness and disease status

Clinicopathologic features	RAI+/D+ (23)	RAI−/D+ (32)	RAI+/D- (15)	P
Age at diagnosis (years), median ± SD	44 ± 20.5	47 ± 18.7	44 ± 16.6	0.81
Female gender, n (%)	16 (70)	21 (66)	10 (67)	0.95
Tumor diagnosis, n (%)				
Incidental	4 (17)	5 (16)	1 (7)	0.62
Pre-surgical	19 (83)	27 (84)	14 (93)	
Tumor size (mm), median (IQR)	2.3 (0.9–0.6)	2.2 (0.7–4.2)	2.4 (1.2–5)	0.27
Histological variant, n (%)				
CPTC	19 (83)	29 (90)	10 (67)	0.001
FVPTC	0	3 (10)	5 (33)	
SCL/COL/TALL/PDTC	2/0/1/1 (9/0/4/4)	0/0/0/0	0/0/0/0	
Extrathyroidal Invasion, n (%)				
Yes	18 (78)	18 (56)	10 (67)	0.23
No	5 (22)	14 (44)	5 (33)	
Multifocality, n (%)				
Yes	16 (70)	17 (53)	12 (80)	0.16
No	7 (30)	15 (47)	3 (20)	
TNM, n (%)				
T1	3 (13)	8 (25)	2 (13)	0.28
T2-T3-T4	20 (87)	24 (75)	13 (87)	
N0	0 (0)	6 (19)	0 (0)	0.24
N1	23 (100)	26 (81)	15 (100)	
M0	18 (78)	25 (78)	15 (100)	0.13
M1	5 (22)	7 (22)	0	
AJCC stage, n (%)				
I	13 (57)	15 (47)	9 (60)	0.64
II-III-IV	10 (43)	17 (53)	6 (40)	
ATA risk stratification, n (%)				
Low	0 (0)	0 (0)	0 (0)	0.61
Intermediate/High	22/1 (96/4)	30/2 (94/6)	15/0 (100/0)	
Cumulative dose of RAI (mCi) median ± SD	85 ± 47.8	76 ± 37.1	75 ± 33.4	0.21

All cases have been submitted to radioiodine ablation after surgery

*Legend*: *RAI* radioiodine, *RAI-* without radioiodine uptake, *RAI+* with radioiodine uptake, *D* disease, *D-* cured, *D+* persistence/relapse, *SD* Standard Deviation, *CPTC* Conventional Papillary Thyroid Carcinoma, *FVPTC* Follicular Variant Papillary Thyroid Carcinoma, *SCL* Sclerosing Papillary Thyroid Carcinoma, *COL* Columnar Papillary Thyroid Carcinoma, *TALL* Tall Cell Papillary Thyroid Carcinoma, *PDTC* Poorly Differentiated Thyroid Carcinoma

According to patients’ classification, RAI-R patients (RAI+/D+, RAI−/D+) were in disease persistence at the last follow-up, while RAI-A patients (RAI+/D-) were all in remission after radiometabolic treatment. In addition, the two groups of RAI-R patients had comparable prevalence of both disease progression (RAI+/D+, 3/23 (13%) vs. RAI−/D+, 8/32 (25%)) and stable disease persistence (RAI+/D+, 20/23 (87%) vs. RAI−/D+, 24/32, (75%)) (*P* = 0.278).

### The molecular profile of PTCs correlated to radioiodine resistance

#### Analysis in 70 primary PTCs

The molecular profile was different in the 3 groups of patients: *BRAF*^*V600E*^ was significantly more frequent in RAI−/D+ group (66%) compared with RAI+/D+ (35%) and RAI+/D- (33%) groups (*P* = 0.032). Consistent with previous findings on the association between *RAS* mutations and RAI-avidity, *RAS* mutations were identified in RAI+/D- (7%), and in the RAI+/D+ group (9%), but not in the RAI−/D+ group (*P* = 0.82, Fig. 2).

The prevalence of the other genetic alterations was not significantly different among the 3 groups, but it is worth to note that, though not statistically significant, probably due to the small number of cases, fusion genes (in particular *RET/PTC*) were more frequent in RAI+/D+ (39%), compared with RAI−/D+ (16%) and RAI+/D- (13%) patients (*P* = 0.075). *TERT* promoter (*pTERT*) mutations had a higher prevalence, though not statistically significant, in the group without RAI uptake (RAI−/D+, 28%) compared with the RAI+/D+ and RAI+/D- groups (17 and 20%) (*P* = 0.61).

Finally, the prevalence of PTCs that were wild type for all the analyzed point mutations and fusions was higher in RAI-avid group (RAI+/D-, 40%) than in RAI-refractory PTCs (RAI+/D+ 17% and RAI−/D+ 22%, *P* = 0.41) (Fig. 2).

**Fig. 3:**
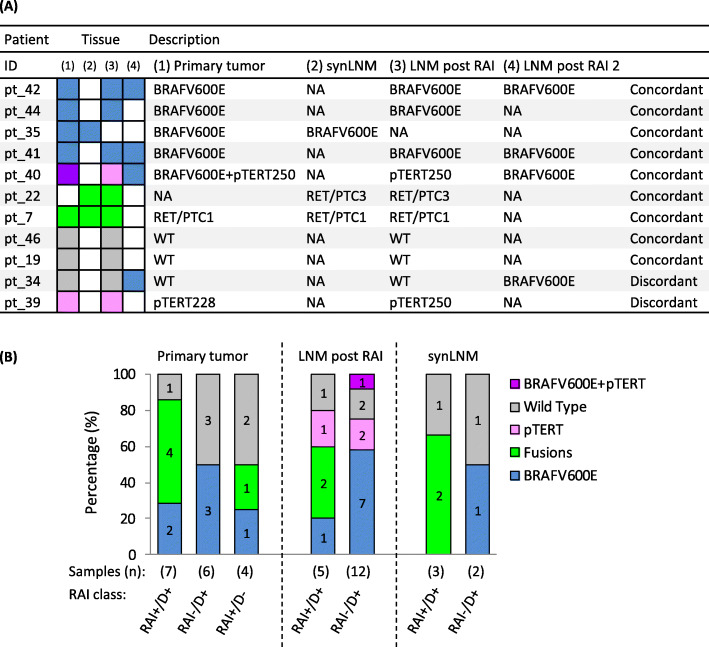
Molecular profiling by PTC-MA in primary and lymph node metastatic PTCs. **a** Driving lesions by PTC-MA in patients with multiple tumor specimens. Color code: light blue, BRAFV600E; green, gene fusions; pink, pTERT mutations; grey, Wild Type (WT) for PTC-MA assay; purple BRAV600E+ pTERT mutation; white, not available (NA) tissue. Detailed mutation/gene fusion description on the right. **b** Driving lesions by PTC-MA in the 39 PTC tissues investigated by gene/miRNA microarrays. Samples were stratified according to tissue type and patient RAI class. Data are shown as percentages; the corresponding sample numbers are indicated in the bars. The total number of samples for each class is indicated in brackets. Abbreviations: RAI, radioiodine; synLNM, synchronous lymph node metastasis; LNM post RAI, lymph node metastasis diagnosed and collected after RAI; RAI+/D+, RAI uptake at the metastatic site and disease persistence; RAI−/D+, no RAI uptake at the metastatic site and disease persistence; RAI+/D-, RAI uptake at the metastatic site and disease remission

In 9/11 patients with available multiple tumor tissues we found concordant genotypes (Fig. 3a), with either *BRAF*^*V600E*^ mutations or gene fusions detected both at the primary tumor and at the metastatic sites.

No statistically significant differences were found in the allelic frequencies of point mutations among the 3 groups analyzed: the mean ± SD normalized allelic frequencies were 23.3 ± 10.1 in RAI+/D+, 30 ± 9.2 in RAI−/D+ and 35 ± 9.4 in RAI+/D- for *BRAF* (*P* = 0.13); 37 ± 15.1 in RAI+/D+, 41.5 ± 10.2 in RAI−/D+ and 47 ± 32 in RAI+/D- for *pTERT* (*P* = 0.37) and 33 ± 1.4 in RAI+/D+ and 32 in RAI+/D- for *RAS* mutations.

#### Analysis in the subgroup for gene/miRNA studies

The 39 tumor samples that underwent gene/miRNA analysis were also assessed by PTC-MA profiling (Fig. 3b). They included both primary (*n* = 17) and lymph node metastases (*n* = 22, 5 collected before and 17 after RAI treatment). Of note, the 17 primary tumors included in this set displayed a genotype-RAI class distribution, with *BRAF*^*V600E*^ mutation more frequent in RAI−/D+ patients, and fusion genes in RAI+/D+ patients, similar to that observed in the 70 primary PTCs (Fig. 2), thus representing a reliable subgroup.

In LNMs collected after RAI (LNM post RAI) *BRAF*^*V600E*^ mutation was more frequent in RAI−/D+ (58% vs. 20%, *P* = 0.14), while fusion genes in RAI+/D+ patients (40% vs. 0%, *P* = 0.09), consistently with the results found in primary PTCs. A similar enrichment was observed also in synLNMs, even though only few samples were tested. Interestingly, *pTERT* mutations were detected only in LNMs post RAI, while no *RAS* mutations were found (Fig. 3b). Overall, driving genetic alterations were identified in 28 (72%) of the 39 tumor tissues.

### Gene/miRNA expression in RAI-refractory and RAI-avid PTCs

Gene and miRNA expression was thus investigated in the above described series of 39 samples, including primary PTCs and metastatic LN, and in 13 available matched NTs, included as control (Fig. 1).

First, by using a TCGA derived gene signature [30] we tested the BRAF−/RAS-related signaling and confirmed that *BRAF*^*V600E*^ mutated tumors, independently of the tissue type, were BRAF-like, as well as most of the detected gene fusions, and that both BRAF- and RAS-like subtypes were present in WT samples (Additional file 1: Suppl. Figure S1).

Considering gene profiles, we found, as expected, a significant separation between tumor and NT tissues, both via unsupervised hierarchical clustering and principal component analysis (PCA; Additional file 1: Suppl. Figure S2A and E). Focusing on PTCs, we did not observe a clear separation considering either tumor tissue type (Additional file 1: Suppl. Figure S2A and E), or collection before or after RAI treatment (Additional file 1: Suppl. Figure S2B), or patient RAI class (Additional file 1: Suppl. Figure S2B and S2F), or driving lesion (Additional file 1: Suppl. Figure S2C), but tumor samples were distinctly separated in two major clusters (Tumor cluster 1 and 2, Additional file 1: Suppl. Figure S2A). The two groups had a different tissue composition in term of cancer cells content. Different tumor purity was indeed found by the gene expression based algorithm CIBERSORT (Additional file 1: Suppl. Figure S2D and G) and by tissue histological review (Additional file 1: Suppl. Figure S2H), that confirmed in low purity tissues a reduced cancer cell content and increased presence of microenvironment derived cells. Interestingly, low tissue purity was observed also in a subgroup of NTs (NT 2 cluster; Additional file 1: Suppl. Figure S2D and G); clinical records and histological revision confirmed the presence of an autoimmune thyroiditis in these patients (Additional file 1: Suppl. Figure S2I). Of note, also the primary tumors included in the low purity group (Tumor cluster 2) displayed at histology a more frequently associated thyroiditis (Tumor cluster 2, 63%, vs. Tumor cluster 1, 11%, *P* = 0.026¸ Additional file 1: Suppl. Figure S2I).

As in gene expression studies reduced tumor cell content can negatively affect the analysis of clinical tumor specimens, since the presence of infiltrating cells can cause the dilution of tumor-specific mRNAs and miRNAs, we decided to focus only on high-purity tissues (based on CIBERSORT class; Additional file 1: Suppl. Figure S2D and G). In addition, as these latter included only two PTCs from RAI-avid patients and could be only partially representative of this group, we excluded these two samples from the subsequent analyses and focused on RAI-refractory cases, as below described.

### Gene/miRNA expression in RAI-refractory primary and metastatic PTCs

Gene/miRNA expression was thus specifically reanalyzed in RAI-refractory patients (RAI+/D+ and RAI−/D+), comprising a total of 29 tissues (10 primary and 10 metastatic LN, and 9 NTs; Fig. 1).

**Fig. 4:**
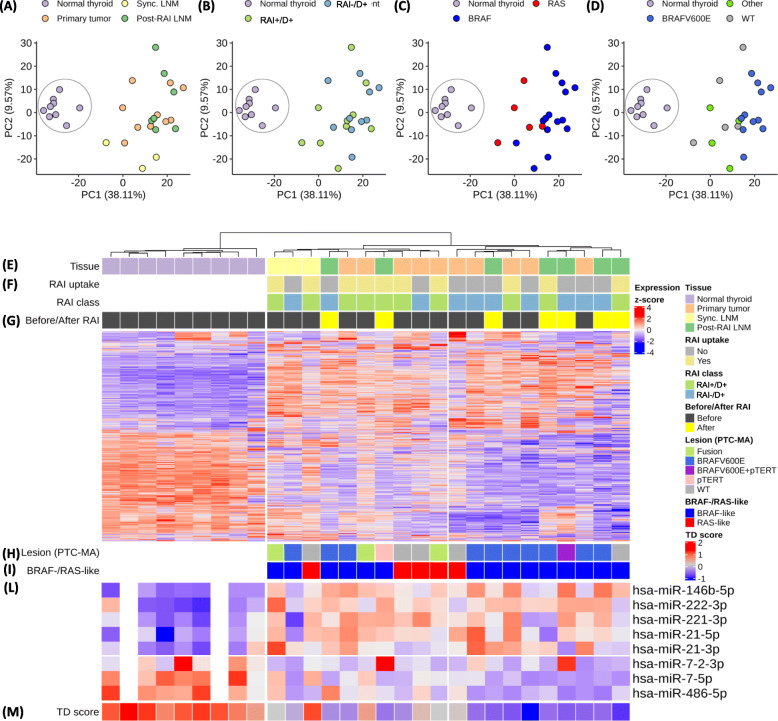
Gene/miRNA expression in RAI-refractory PTCs. Principal component analysis (PCA) using the top most variable genes according to inter-quartile range. Specific samples features are showed separately: **a** Tissue type; **b** RAI class; **c** BRAF−/RAS-like subtype; **d** Driving lesion by PTC-MA; class Other includes 3 samples with gene fusions and 1 with pTERT mutation. Normal thyroid group is highlighted by a circle. Unsupervised hierarchical clustering of the top most variable genes according to inter-quartile range; Euclidean distance and Ward linkage. The following samples features are showed: **e** Tissue type. **f** RAI uptake and class. **g** Collection before and after RAI. **h** Driving lesion by PTC-MA. **i** BRAF−/RAS-like subtype. **l** Significantly deregulated miRNA, expression row Z-score; for 2 NT samples, depicted in white, the corresponding miRNA profiles were not available. **m** Thyroid differentiation (TD) score calculate as mean expression across 16 thyroid function related genes derived from TCGA. Abbreviations: RAI, radioiodine; LNM, lymph node metastasis; synLNM, synchronous lymph node metastasis; RAI+/D+, RAI uptake at the metastatic site and disease persistence; RAI−/D+, no RAI uptake at the metastatic site and disease persistence

Considering gene expression, we confirmed also in this set a significant separation between tumor and NT (Fig. 4a and e). No clear separation was found between the different tumor tissues (Fig. 4a), even though the majority of LNMs post RAI (5/7, 72%) localized in the same cluster (Fig. 4e). No significant separation was observed according to patient RAI-refractoriness class (Fig. 4b and f). On the other hand, a significant stratification was observed according to the BRAF−/RAS-subtype (Fig. 4c) and to the driver genetic alteration (Fig. 4d). In particular, RAI-refractory *BRAF*^*V600E*^ tumors appeared as a quite homogenous group (Fig. 4d) with the majority of samples clustering together (8/11, 73%; Fig. 4h) regardless of either the tissue type (i.e. primary tumors and LFN post RAI; Fig. 4e) or the collection before or after RAI treatment (Fig. 4g).

Similar results were obtained by miRNA profiles. Significant miRNA deregulation was observed in PTCs compared with NTs, confirming the upregulation of broadly reported oncogenic miRNAs (miR-146b-5p, miR-222-3p, miR-221-3p, miR-21-5p/−3p) and the downregulation of miR-7-2-3p/−5p and miR-486-5p (Fig. 4l). However, no significant differences were found in their expression across tumor samples either based on tissue type, or patient RAI class or collection before or after RAI treatment (Fig. 4e-g). Along with these, we confirmed the up- and down-modulation of additional miRNAs (Additional file 1: Suppl. Figure S3), whose deregulation has been already reported in PTC (revised in [24]). Considering these PTC related miRNAs (Additional file 1: Suppl. Figure S3C), we observed samples stratification according to *BRAF*^*V600E*^ mutation (Additional file 1: Suppl. Figure S3D) and BRAF−/RAS-like subtype (Additional file 1: Suppl. Figure S3E). Interestingly, we found that while miRNA upregulation was consistent across the various driving lesions and BRAF−/RAS-subtypes, miRNA downregulation was more evident in *BRAF*^*V600E*^ and in BRAF-like tumors (Additional file 1: Suppl. Figure S3H-I).

In our series of RAI refractory PTCs along with miRNA, we found consistent gene deregulation. Many genes were differentially expressed in PTCs compared with NTs (Additional file 1: Suppl. Figure S4A), and their deregulation was confirmed in the large PTC series from TCGA (Additional file 1: Suppl. Figure S4B). Upregulated genes were associated with cell junction/adhesion, integrin and extracellular matrix remodeling, and inflammation (Additional file 1: Suppl. Figure S5A); they included also MAPK pathway regulators (Additional file 1: Suppl. Figure S5B) and genes of BRAF−/RAS-like subtype signature. Downregulated genes were involved in metabolic processes, and, more interestingly, included pendrin (*SLC26A4*), thyroid peroxidase (*TPO*) and several other genes related to thyroid function and differentiation (Additional file 1: Suppl. Figure S5C-D).

**Fig. 5:**
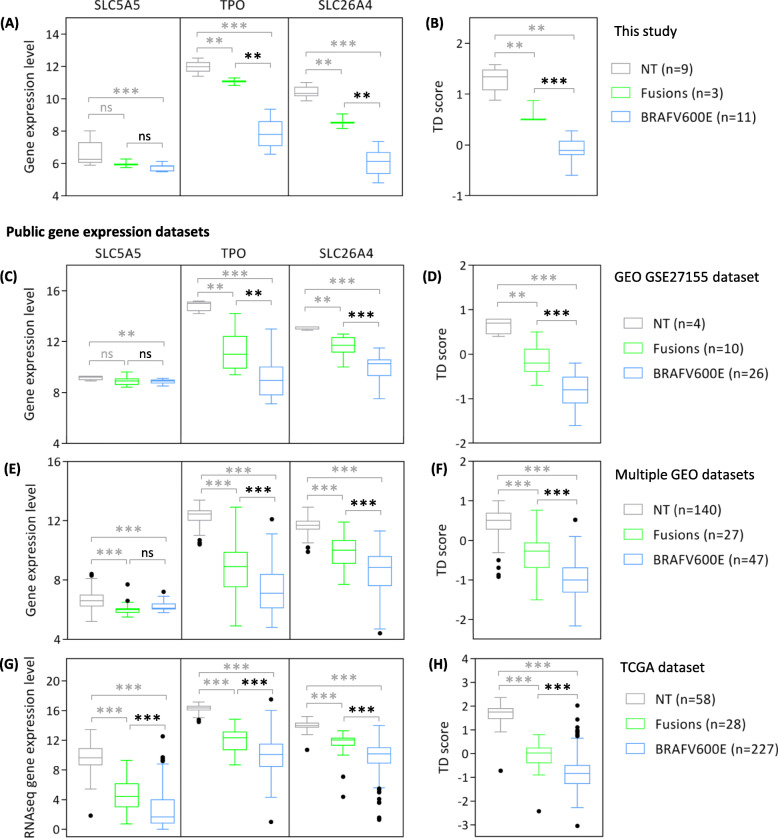
Thyroid differentiation gene expression in PTCs stratified for *BRAF*^*V600E*^ mutation and gene fusions. The expression of *NIS* (*SLC5A5*), thyroperoxidase (*TPO*) and pendrin (*SLC26A4*) genes is shown in PTCs and normal thyroid (NT) samples derived from the present study (**a**), from GEO dataset GSE27155 (**c**), from multiple GEO datasets (**e**) and from TCGA (**g**). Normalized gene expression values (Log2 scale) are showed for microarray studies (**a**), (**c**) and (**e**); RNAseq normalized expression values (Log2 scale) are showed for TCGA series (**g**). Thyroid differentiation (TD) score has been calculated for each samples series (**b**), (**d**), (**f**) and (**h**) as mean expression across the median-centered expression of 16 thyroid function related genes derived from TCGA (see material and methods). Only data for PTC samples with reported *BRA*^*V600E*^ mutation or *RET* or *NTRK1* gene fusions are showed. * *p*-value < 0.05 ** *p*-value < 0.005; *** *p*-value < 0.0001 by Mann-Whitney test

As the expression of *NIS* (*SLC5A5*), *TPO* and *SLC26A4* has been already proposed to affect RAI uptake and response, we tested whether their expression could be different in our PTC samples stratified for RAI class of refractoriness (Additional file 1: Suppl. Figure S6A). Interestingly, a significant difference was observed for *SLC26A4* gene. Moreover, we tested the possible association between gene expression and molecular profile of our RAI-refractory cases. We found that *TPO* and *SLC26A4* genes were consistently different between *BRAF*^*V600E*^ and gene fusions positive tumors (Fig. 5a), while, by contrast, *NIS* expression appeared to be similarly reduced. This was confirmed in other PTC series derived from public repositories (GEO datasets, Fig. 5c and e; and TCGA dataset, Fig. 5g), where significant differences were observed for *TPO* and *SLC26A4* genes, whereas *NIS* expression was significantly reduced only in TCGA dataset. Along with these, other genes related to thyroid metabolism and function exist and TCGA proposed a Thyroid Differentiation (TD) score [30] that summarizes the expression of 16 thyroid related genes (also including the above mentioned *SLC5A5*, *TPO*, and *SLC26A4*). We applied the TCGA established TD score and not only confirmed its expression in PTC tissues (Additional file 1: Suppl. Figure S6C), but also its high level in normal thyroid, which was unexplored for TD score in TCGA study (Additional file 1: Suppl. Figure S6D). The same TD score was applied to our samples series (Additional file 1: Suppl. Figure S6E). While no significant differences were observed according to RAI refractoriness class (Additional file 1: Suppl. Figure S6B), it was significantly different in *BRAF*^*V600E*^ compared to gene fusions, with *BRAF*^*V600E*^ PTCs displaying reduced thyroid differentiation levels (Fig. 5b). The same results were obtained in public datasets (Fig. 5d, f and h). In addition, TD score was significantly associated with sample clustering observed in both gene and miRNA expression profiles (Fig. 4m and Additional file 1: Suppl. Figure 3G).

## Discussion

In this study we investigated the molecular profiles and gene/miRNA expression in metastatic PTC patients stratified for RAI response in accordance with their RAI-refractoriness/avidity.

By the PTC-MA platform we found a different molecular profile in RAI-avid and RAI-refractory PTCs. *BRAF*^*V600E*^ was significantly more frequent in PTCs unable to uptake RAI at the metastatic site already at the time of the diagnosis. An association between *BRAF* mutation and RAI-R disease has been previously reported [4–6, 9], though a whole molecular profile was not obtained and cases were not classified according to the “type” of RAI refractoriness, evident at the time of initial treatment or developed during time. Our data indicate that *BRAF*^*V600E*^ associates with an intrinsic tumor RAI refractoriness, being significantly more prevalent in tumors RAI−/D+. On the contrary, we report for the first time a higher prevalence of fusion genes in PTCs with initial RAI uptake, but without therapeutic efficacy (RAI+/D+), suggesting that also these molecular alterations interfere with the iodine organification process, though not impairing iodine uptake. Accordingly, the partial response recently reported in an advanced DTC harboring an *EML4-NTRK3* fusion and treated with Larotrectinib, a NTRK inhibitor, is consistent with the restoration of radioiodine organification [44]. *pTERT* mutations were more frequent in the PTC group without RAI-uptake, in accordance with data that report for this genetic alteration a negative prognostic role in thyroid cancer [45]. On the other hand, cases wild type for all the tested mutations and gene fusions were more prevalent in patients with RAI-avid metastatic disease in remission, indicating that gene alterations not analyzed by our assay are more frequently associated with a more differentiated and less aggressive disease.

In patients with available multiple tumor specimens, we found in most cases concordant genetic patterns between primary PTC and lymph node metastases, not only for *BRAF*^*V600E*^ mutation, in agreement with previous reports [9], but also for *RET/PTC* fusions. This was observed not only in synchronous LNMs but also in RAI-refractory LNM collected after RAI treatment.

The mutational profiles of primary and synchronous metastases in thyroid cancers, including PTC, have been already investigated [46–50], describing high and significant concordance between the primary tumor and the corresponding metastasis, especially the LNM. On the other hand, only few studies assessed the global gene/miRNA expression in these tissue types [43, 51, 52], confirming transcriptome similarity between primary TC and matched synchronous LNMs. To our knowledge, this is the first study investigating gene/miRNA expression in PTC tissues including RAI-refractory LNMs collected following RAI treatment.

We initially investigated a series including primary and LN metastatic PTCs, from either RAI-refractory or -avid patients. The gene profiles identified a clear separation between neoplastic and normal tissues, and tumor samples were also found to stratify into 2 major clusters, according to the tissues composition, and in particular to presence or not of an associated autoimmune thyroiditis or other types of immune cells infiltrating the tissue for primary tumors and NTs, and, of residual lymphoid tissue not replaced by neoplastic cells for LNMs. Indeed, PTC tissues including very high or low amount of immune cells displayed a distinguishable transcriptomic profile, which should be further evaluated for possible clinical impact in larger series.

On the other hand, the presence of these tumor infiltrating cells, of various origin, might have affected our analyses impairing a clear identification of PTC-specific mRNAs and miRNAs, as already reported [31, 43, 53–55].

With the aim of identifying tumor related features, that could be not perturbed by the presence of other microenvironment derived cellular components, we thus focused on the subset of samples displaying high tissue purity from RAI-refractory patients.

In this sample set gene profiles confirmed not only the separation between tumors and NTs, but also and more interestingly, the transcriptome similarity among *BRAF*^*V600E*^ mutated samples, either primary or LNMs post RAI, thus suggesting that radioiodine treatment minimally affects the expression profiles of RAI-refractory lymph metastases with respect to primary tumors.

In this samples group we also confirmed the up- and down- regulation of previously reported PTC-related miRNAs (revised in [24]). In addition, we found a correlation with genotype and a more pronounced miRNA downregulation in *BRAF*^*V600E*^ and BRAF-like tumors, suggestive of more dedifferentiated tissues.

To the best of our knowledge this is the first study investigating gene/miRNA expression in tumor samples from RAI refractory patients, and including not only primary PTCs, but also synchronous and RAI-refractory LNMs. We found gene and miRNA deregulations consistent with those described by previous reports, such as the TCGA study [30], which analyzed primary PTCs, regardless of the RAI classification. In addition, according with TCGA [30], that describes how PTC subclassification is influenced by BRAF−/RAS-subtype and by the expression of thyroid related genes (TD score), along with the histological variant and driving lesion, we found that in our series from RAI-refractory patients (RAI+/D+ and RAI−/D+) the major classifying features are still the driving lesion (and in particular *BRAF*^*V600E*^), the BRAF−/RAS-subtype and the TD score.

Among TD genes, *NIS* was found to be significantly dowregulated in RAI-refractory tumors, and particularly in RAI−/D+ cases, consistent with the clinical behavior. As an original finding, also *SLC26A4* was significantly more downregulated in RAI−/D+ than in RAI+/D+ cases, suggesting that these cases are even more dedifferentiated and that if a small amount of RAI is uptaken, it is immediately washed out due to the lack of the apical transporter [56]. Finally, *TPO* was downregulated in both RAI refractory classes, as expected for tissues not able to organify and thus retain iodine into the colloid. By evaluating these crucial genes in relation to the genetic pattern, we found that *NIS* appeared to be similarly reduced in *BRAF*^*V600E*^ and fusions positive tumors, whereas *TPO*, *SLC26A4* and TD score were significantly more downregulated in *BRAF*^*V600E*^ tumors than in fusion positive tumors. Interestingly, these data were further confirmed by analyzing other series from public repositories and are consistent with the already reported dedifferentiation [30] and RAI refractoriness associated with *BRAF*^*V600E*^ mutation [9, 10].

Further studies to get more insights into the functional relationships among *BRAF* and *RET/PTC* oncogenes, RAI uptake and expression of thyroid differentiation genes such as *NIS* and *SLC26A4*, would be useful. In this context it was demonstrated that in mouse thyroid cancers with conditional *BRAF*^*V600E*^ expression the lack of radioiodine incorporation depend on BRAF activity [57]. In the clinical setting, promising data on redifferentiation have been obtained with new compounds targeting BRAF or MEK, such as Vemurafenib, Dabrafenib, Trametinib, and Selumetinib, though the anti-tumor effect is variable, ranging from a partial to a null response, possibly due to the histopathological and genetic heterogeneity of mutated tumors [11–19]. Finally, based on the original data obtained in the present work, future studies will be particularly focused on the possible effects of *RET/PTC* oncogenes on RAI uptake, since to the best of our knowledge no data are available on this topic.

## Conclusions

We studied the molecular profile and gene/miRNA expression in primary PTCs, synchronous and RAI-refractory LNMs in correlation to RAI avidity or refractoriness.

*BRAF*^*V600E*^ tumors were found more frequently unable to uptake RAI at the metastatic site already at the time of the diagnosis, suggesting an intrinsic RAI refractoriness. Accordingly, the genes related to RAI refractoriness, e.g. those responsible for iodine apical transport or organification, were highly downregulated, regardless of either the different tissue type (i.e. primary tumor, or synchronous or RAI refractory lymph nodal metastases) or the collection before or after RAI treatment, indicating that the dedifferentiation related to this mutation is present at the very early stages of tumor growth. Moreover, a transcriptome similarity was found among *BRAF*^*V600E*^ mutated samples, either primary or lymph nodal metastases post RAI, thus suggesting that radioiodine treatment minimally affects the expression profiles of RAI-refractory lymph metastases with respect to primary tumors.

On the other hand, genes related to RAI refractoriness were less downregulated in fusions positive tumors which were, consistently, more frequently able to uptake RAI, but still with persistent disease, suggesting different mechanisms leading to RAI-refractoriness.

## Supplementary Information

**Additional file 1: Suppl. Figure S1.** Tumor samples driving lesion by PTC-MA and BRAF-/RAS-like subtype. **Suppl. Figure S2.** Gene expression in RAI-refractory and RAI-avid primary and LN metastatic PTCs. **Suppl. Figure S3.** Expression of deregulated miRNAs in our series of RAI-refractory PTCs. **Suppl. Figure S4.** Differentially expressed genes in our series of RAI-refractory PTCs. **Suppl. Figure S5.** Expression of selected genes according to the most deregulated pathways identified in our series of RAI-refractory PTCs. **Suppl. Figure S6.** Thyroid differentiation (TD) genes expression.

**Additional file 2.** Supplemental Methods.

## Data Availability

The datasets generated and/or analysed during the current study are available in the NCBI Gene Expression Omnibus (GEO) repository (www.ncbi.nlm.nih.gov/geo/) with the accession number GSE151179. Gene microarray data include 52 thyroid tissues (39 primary and LN metastatic PTCs and 13 NTs); the corresponding miRNA data were available for 47/52 samples.

## Abbreviations

DTC: Differentiated thyroid cancer
GEO: Gene Expression Omnibus
LNMs: Lymph node metastases
LNM post RAI: Radioiodine-refractory LNM diagnosed and collected after RAI treatment
NIS: Sodium/iodide symporter
NT: Non-neoplastic thyroid
PTC: Papillary thyroid cancer
PTC-MA: PTC-Mass Array
RAI: Radioiodine
RAI-/D+: Absent RAI uptake/disease persistence
RAI+/D-: RAI uptake/disease remission
RAI+/D+: RAI uptake/disease persistence
RAI-A: Radioiodine-avid
RAI-R: Radioiodine-refractory
synLNM: Synchronous lymph node metastasis
TCGA: The Cancer Genome Atlas
TD: Thyroid differentiation
TPO: Thyroperoxidase
