# Supplementary material for: The molecular and gene/miRNA expression profiles of radioiodine resistant papillary thyroid cancer
Source: J Exp Clin Cancer Res. 2020 Nov 16;39:245. doi: 10.1186/s13046-020-01757-x (PMC7667839; doi:10.1186/s13046-020-01757-x)
Supplement: Supplementary file 1 — Additional file 1: Suppl. Figure S1. Tumor samples driving lesion by PTC-MA and BRAF-/RAS-like subtype. Suppl. Figure S2. Gene expression in RAI-refractory and RAI-avid primary and LN metastatic PTCs. Suppl. Figure S3. Expression of deregulated miRNAs in our series of RAI-refractory PTCs. Suppl. Figure S4. Differentially expressed genes in our series of RAI-refractory PTCs. Suppl. Figure S5. Expression of selected genes according to the most deregulated pathways identified in our series of RAI-refractory PTCs. Suppl. Figure S6. Thyroid differentiation (TD) genes expression. [file 13046_2020_1757_MOESM1_ESM.pdf]

**Figure S1**

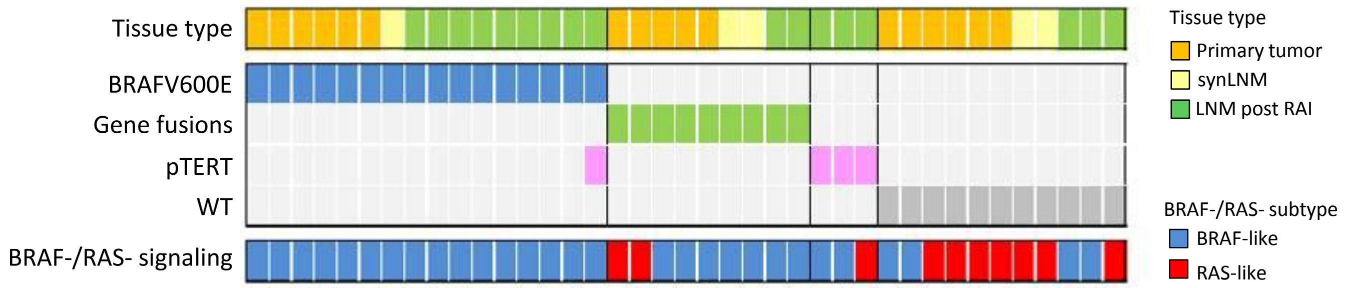


**Figure S1**. **Tumor samples driving lesion by PTC-MA and BRAF-/RAS-like subtype**.
Gene driver by PTC-MA and BRAF-/RAS-like subtype in the 39 tumor samples investigated by gene and miRNA microarrays. The BRAF- or RAS-like class was assigned based on the 71-gene signature derived from TCGA.
Abbreviation: LNM, lymph node metastasis; synLNM, synchronous lymph node metastasis

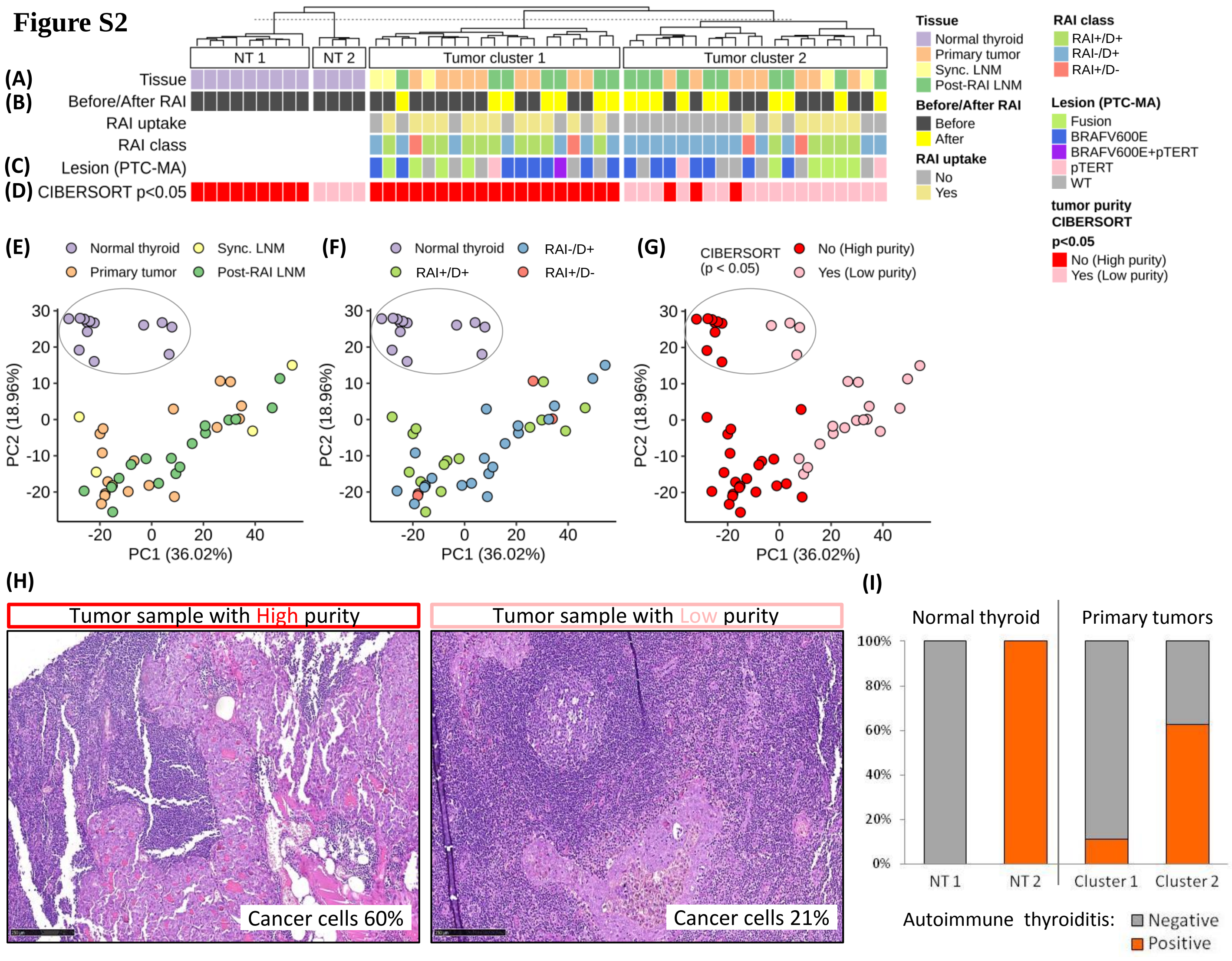


**Figure S2. Gene expression in RAI-refractory and RAI-avid primary and LN metastatic PTCs.** Unsupervised hierarchical clustering of the top most variable genes according to inter-quartile range; Euclidean distance and Ward linkage. Two major clusters were identified for tumor (Tumor cluster 1 and 2) and normal thyroid tissues (NT1 and 2). The following samples features are showed: **(A)** Tissue type; **(B)** Collection before and after RAI, RAI uptake and class; **(C)** Driving lesion by PTC-MA; **(D)** Tumor purity calculated by CIBERSORT algorithm; class attribution according to p-value (p-value < 0.05, low purity; p-value ≥ 0.05, high purity).

Principal component analysis (PCA) using the top most variable genes according to inter-quartile range. Specific samples features are showed separately: **(E)** Tissue type; **(F)** RAI class; **(G)** Tumor purity class by CIBERSORT. Normal thyroid group is highlighted by a circle.

**(H)** Hematoxylin and eosin sections of two representative tumor specimens included in Tumor cluster 1 and 2 displaying high and low tumor purity, respectively. The corresponding cancer cell percentage established by histological revision is indicated. Both tissues are derived from lymph node metastases. Scale bar 250µm. **(I)** Samples distribution based on the presence of autoimmune thyroiditis in high (NT1 and Cluster1) and low purity (NT2 and Cluster2) gene clusters reported in **(A).**

Abbreviations: RAI, radioiodine; LNM, lymph node metastasis; synLNM, synchronous lymph node metastasis; RAI+/D+, RAI uptake at the metastatic site and disease persistence; RAI-/D+, no RAI uptake at the metastatic site and disease persistence; RAI+/D-, RAI uptake at the metastatic site and disease remission.

**Figure S3**

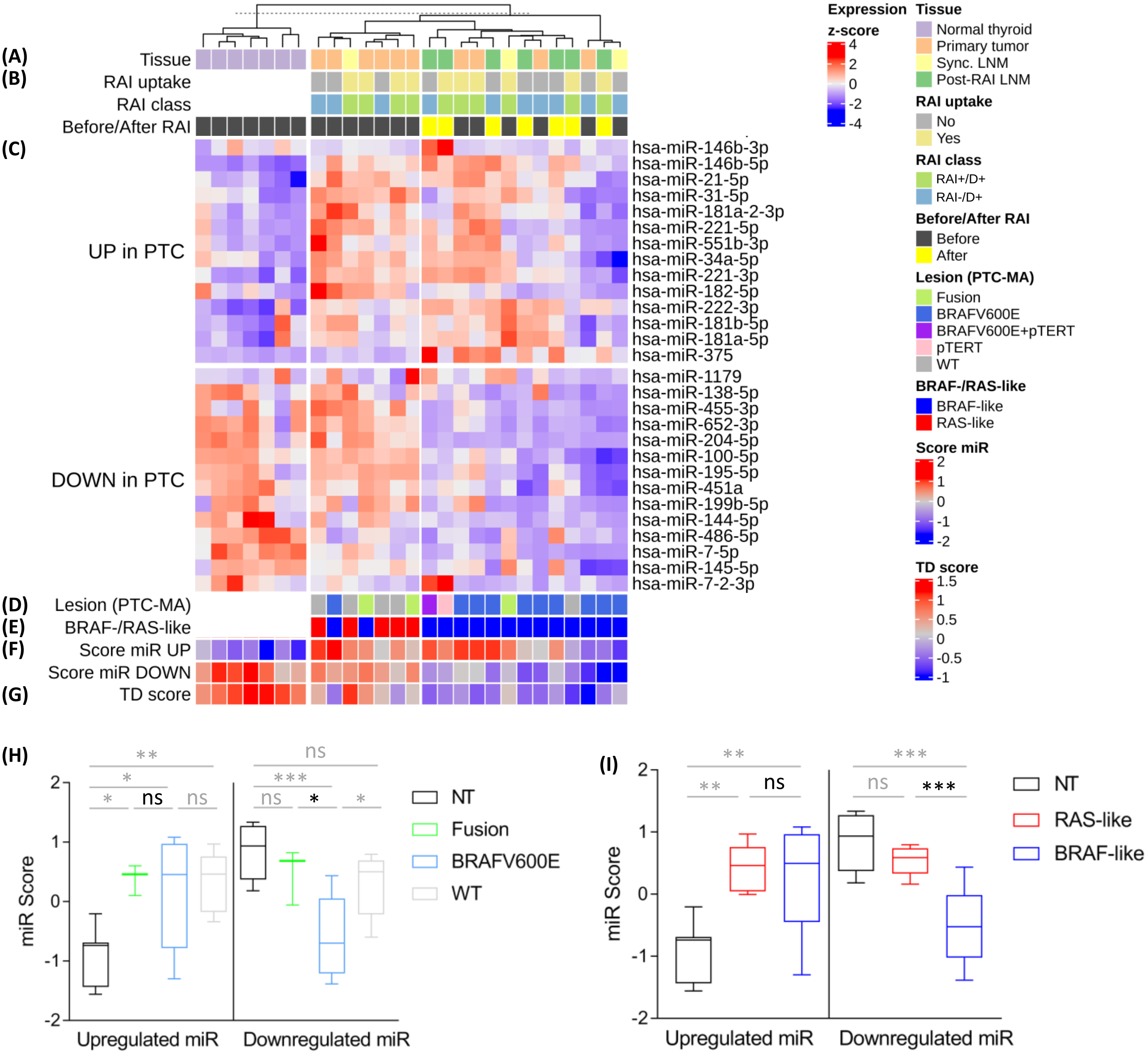


**Figure S3**. **Expression of deregulated miRNAs in our series of RAI-refractory PTCs**.
Unsupervised hierarchical clustering of 28 miRNAs reported in literature as deregulated in PTC; miRNA profiles available for 27/29 samples. The following samples features are showed: **(A)** Tissue type; **(B)** RAI uptake, class and collection before and after RAI treatment; **(C)** miRNA expression, row Z-score; **(D)** Driving lesion by PTC-MA; **(E)** BRAF-/RAS-like subtype; **(F)** miRNA expression score calculated as mean of Log2-transformed and median-centered miR expression; separate scores were calculated for the showed UP- and DOWN-regulated miRNAs; **(G)** Thyroid differentiation (TD) score calculate based on the expression of 16 thyroid function related genes derived from TCGA.
miR score levels in samples stratified for: **(H)** Driving lesion; **(I)** BRAF-/RAS-like subtypes.
* p-value < 0.05 ; ** p-value < 0.005 ; *** p-value < 0.0001 by Mann-Whitney test.
Abbreviation: RAI, radioiodine; LNM, lymph node metastasis; synLNM, synchronous lymph node metastasis; RAI+/D+, RAI uptake at the metastatic site and disease persistence; RAI-/D+, no RAI uptake at the metastatic site and disease persistence.

**Figure S4**

(A)

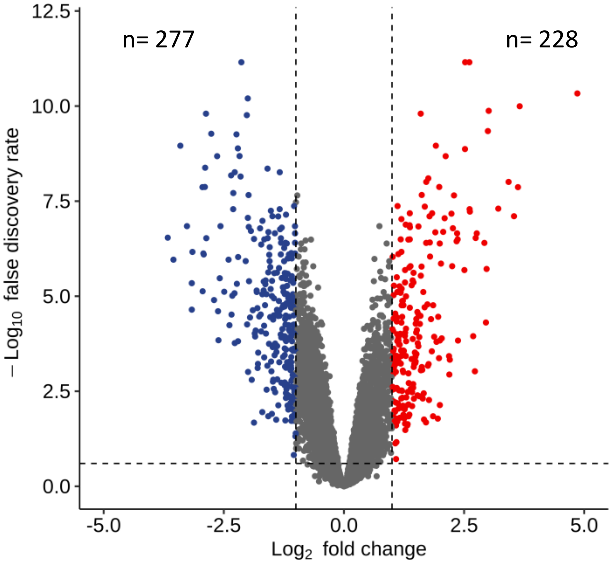


(B)

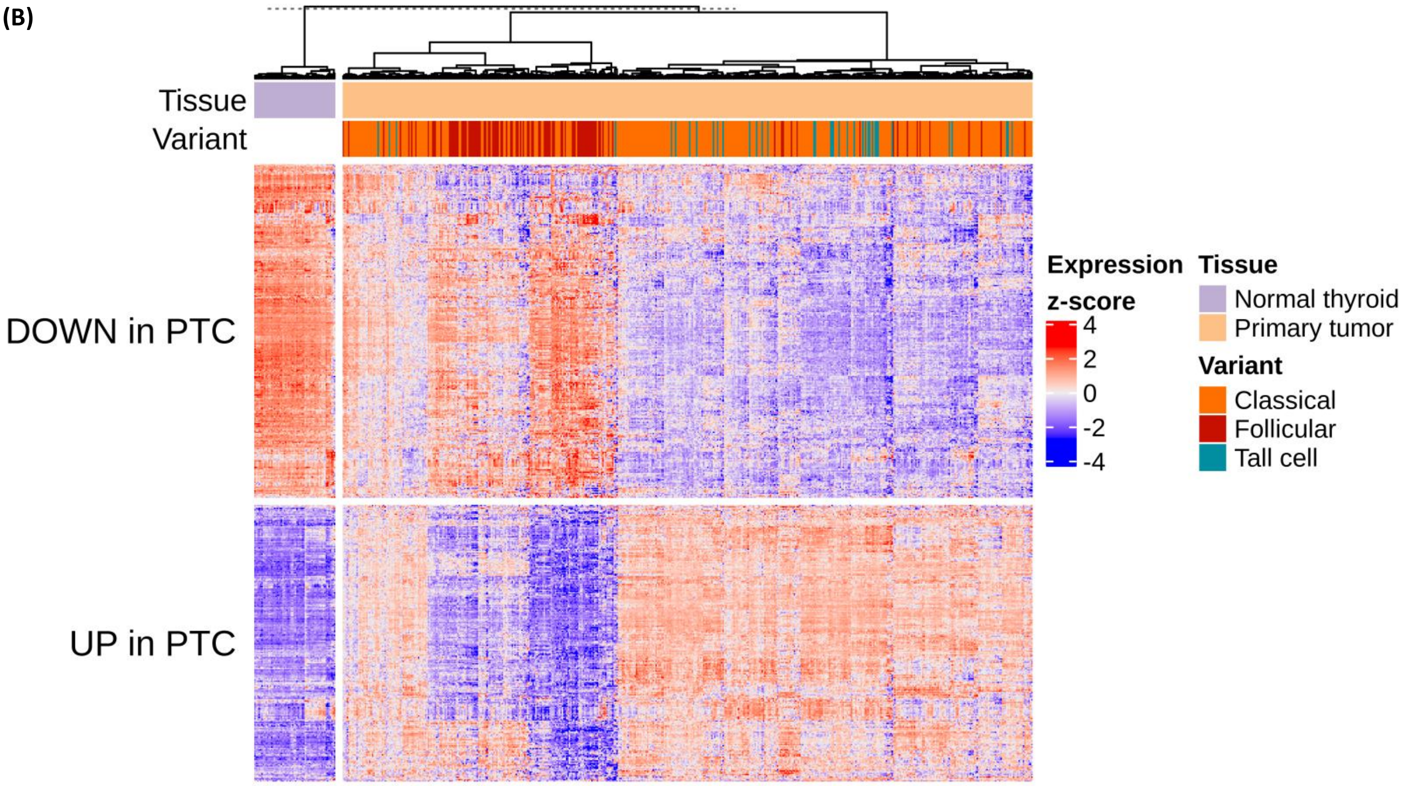


**Figure S4**. **Differentially expressed genes in our series of RAI-refractory PTCs.**
**(A)** Volcano plot of differentially expressed genes identified by class comparison in our sample series of 20 primary and metastatic RAI-refractory PTCs vs. 9 NTs; 505 genes were identified (FDR<0.05; absolute FC 2). Upregulated genes are showed in red, downregulated in blue. **(B)** Unsupervised hierarchical clustering of 494 primary PTCs and 58 NTs from TCGA study, using the list of 505 genes described in (A).

**Figure S5**

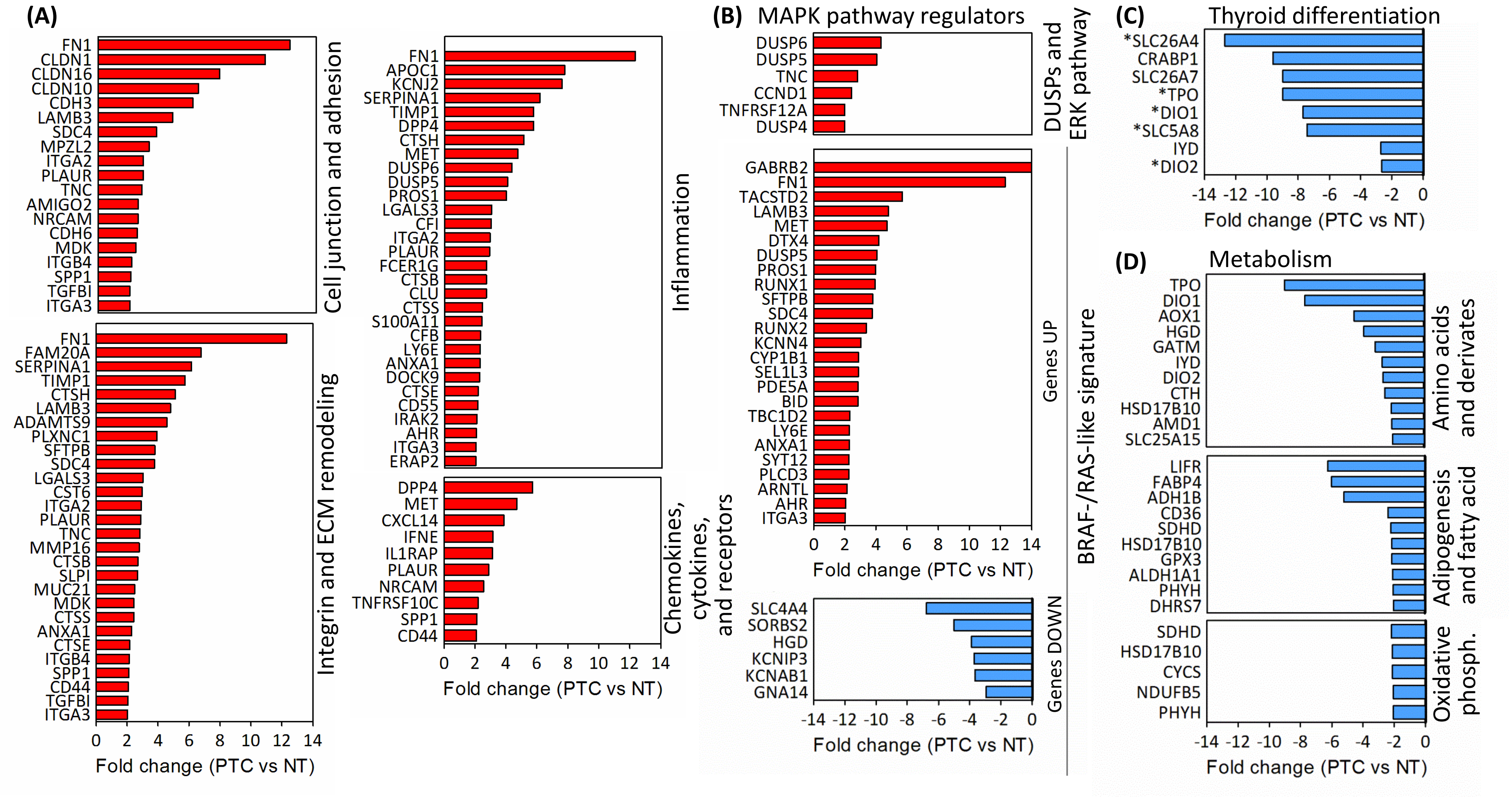


**Figure S5**. **Expression of selected genes according to the most deregulated pathways identified in our series of RAI-refractory PTCs.** Gene expression level is showed as Fold change of the class comparison PTCs (n=20) vs. NTs (n=9). Upregulated genes are showed in red, downregulated genes in blue. Gene pathways were derived from the top significant pathways (FDR <0.05) identified by gene set enrichment analyses using the gene set collections Hallmarks and C2 sub-collection Canonical pathways; additional manually curated gene sets (BRAF-/RAS-like signature and Thyroid Differentiation) were investigated. Only genes included in the set of 505 differentially expressed genes (FDR<0.05; absolute FC 2), are specifically represented.

Abbreviations: RAI, radioiodine; PTC, papillary thyroid carcinoma; NT, non-neoplastic thyroid; ECM, extracellular matrix; Phosph, phosphorylation. * gene included in the Thyroid Differentiation score reported by TCGA (see Suppl.Figure 6)

# Figure S6

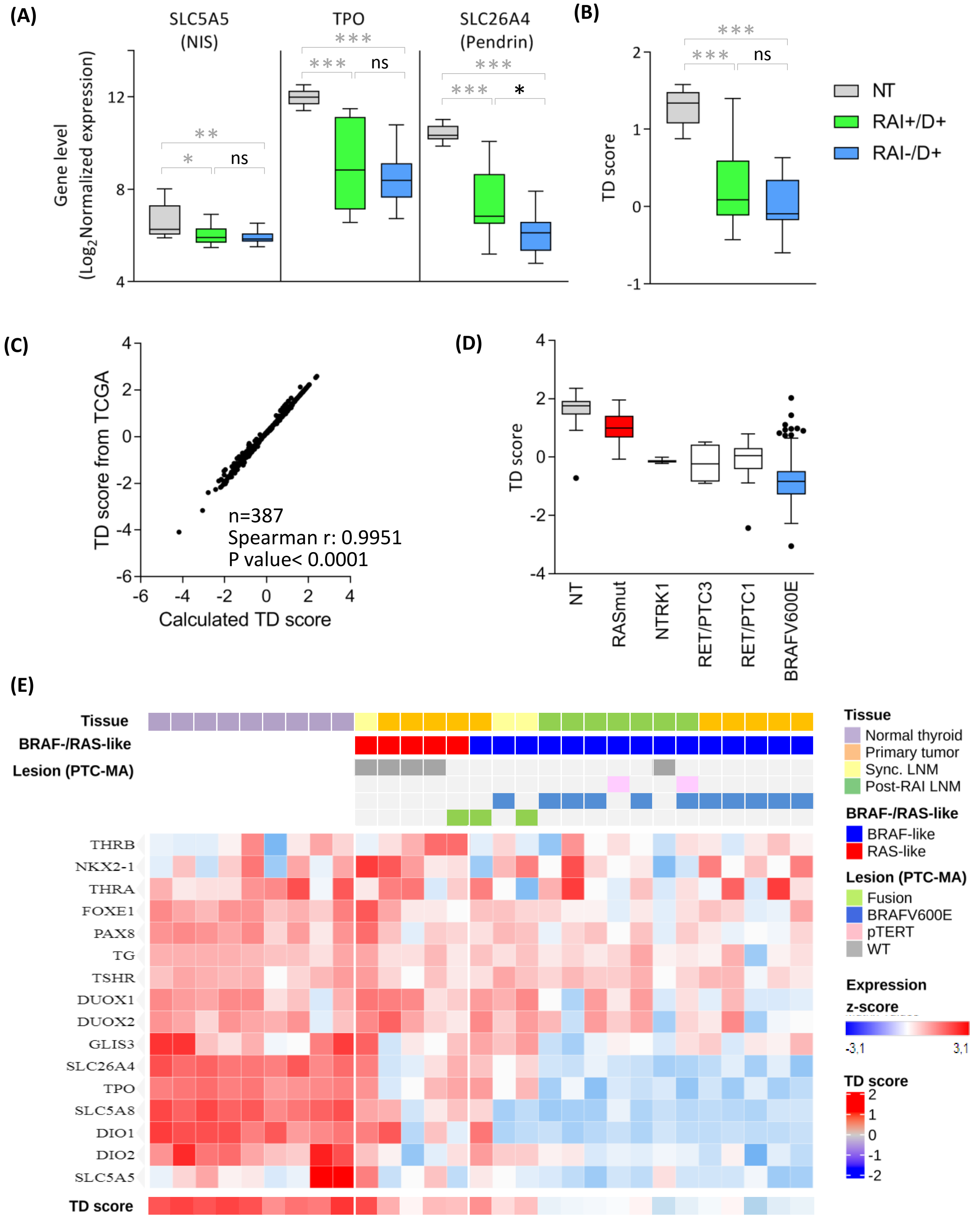


**Figure S6**. **Thyroid differentiation (TD) genes expression**.
**(A)** Gene expression of 3 thyroid function related genes in our series of NTs and RAI-refractory PTCs stratified for RAI class. **(B)** TD score levels in samples reported in (A). **(C)** Correlation between TD score reported in TCGA study and TD score recalculated in the present study for the same series of PTC. **(D)** Calculated TD score levels in PTC and NT samples from TCGA series; only PTC with the indicated driving lesions are showed from the whole series. **(E)** Heatmap showing the 16 TD genes expression and the corresponding calculated TD score in our series of NTs and RAI-refractory PTCs.
* p-value < 0.05 ; ** p-value < 0.005 ; *** p-value < 0.0001 by Mann-Whitney test.
