# Supplementary material for: The molecular and gene/miRNA expression profiles of radioiodine resistant papillary thyroid cancer
Source: J Exp Clin Cancer Res. 2020 Nov 16;39:245. doi: 10.1186/s13046-020-01757-x (PMC7667839; doi:10.1186/s13046-020-01757-x)
Supplement: Supplementary file 2 — Additional file 2. Supplemental Methods. [file 13046_2020_1757_MOESM2_ESM.docx]

**SUPPLEMENTAL MATERIALS AND METHODS**

**Gene/miRNA microarray analysis**

Genomic DNA and RNA were extracted from the collected 69 frozen tissues by Qiagen commercial kits (DNeasy Blood & Tissue Kit and miRNeasy Mini Kit, respectively). DNA and RNA integrity was assessed using Agilent 2100 Bioanalyzer and their quality was evaluated by the DNA and RNA Integrity Number (DIN and RIN, respectively); DNA was high-quality (mean DIN 7.0, range 6.0–8.1) while RNA was of very variable quality (mean RIN 5.0, range 1.0–7.8) with 20% of samples displaying RIN<3, indicative of strongly degraded RNA. RNA quality was further assessed by the DV200 parameter (RNA fragment distribution value) confirming for 11 samples low-quality RNA; these samples were excluded from the subsequent analyses.

Gene profiles were established by Thermo Fisher Human Clariom S Assay. RNA labeling, processing, and hybridization were performed according to manufacturer’s instructions, and microarrays were scanned with the Gene Chip System 3000 scanner. Raw data were pre-processed using the robust multi-array average method (1) implemented in oligo Bioconductor package. Following gene arrays quality control 6 samples resulted low-quality and were excluded from the subsequent analyses. Probe sets were mapped to gene symbols using the annotateEset function of the affycoretools package; probes not associated to gene symbols were filtered out and those mapping to the same gene were collapsed using the collapseRows function of the WGCNA package with “maxRowVariance” method (2).

MiRNA profiles were established by Agilent SurePrint Human miRNA microarrays. RNA labeling, processing, and hybridization were performed according to manufacturer’s protocol, and microarrays were scanned with Agilent SureScan Microarray Scanner. Raw data were pre-processed using the limma package (3). Briefly, raw data were background-corrected using the “normexp” method and quantile-normalized. Probes with intensity lower than at least 10% of the 95^th^ percentile of the negative control probes in all samples were filtered-out. Replicated probes were collapsed by calculating the average expression and probe mapping to the same miRNA were collapsed using the collapseRows function of the WGCNA package with “maxRowVariance” method (2).

Following quality control and data pre-processing, gene expression data were available for 52 thyroid tissues and the corresponding miRNA expression data were available for 47/52 samples. Expression data are deposited and available on NCBI Gene Expression Omnibus (GEO) under the accession number GSE151179.

**Gene expression validation in public datasets**

Human thyroid cancer gene datasets available on public repositories were investigated for genes expression validation.

RNA-Seq raw counts for TCGA study on PTC (4) were downloaded from the Genomic Data Commons data portal (<https://portal.gdc.cancer.gov/>) with accession date 27 April 2020. For hierarchical clustering analysis raw counts data were normalized using the trimmed-mean of M values (5) and transformed in log2 counts per million transcripts using the edgeR package (6); 494 primary PTCs and 58 normal thyroid samples were analyzed.

Additional samples series including PTC and NT were downloaded from GEO and investigated for the expression of thyroid specific genes (*NIS/SLC5A5*, *TPO*, and *SLC24A6*) and a thyroid differentiation (TD) score. Only PTCs with reported *BRAF^V600E^* mutation or *RET* and *NTRK1* gene fusions were specifically studied.

From GSE27155 expression data on Affymetrix U133A array platform were available for 4 NTs and 95 thyroid cancer tissues, including 51 PTCs; among these 26 *BRAF^V600E^* mutated and 10 gene fusion positive (6 with *RET/PTC1*, 3 with *RET/PTC3*, and 1 with a diverse *RET* fusion) PTCs were available. For this set 14/16 TD genes were available for TD score computing.

Additional datasets were investigated (GSE3467, GSE6004, GSE33630, GSE35570, GSE53157, GSE29265, GSE3678, and GSE60542) all profiled on the same microarray platform (Affymetrix Human Genome U133 Plus 2.0 Array). Raw intensity expression values were processed, normalized and batch corrected as previously described (7) to generate a single series of 140 NT and 205 thyroid cancer tissues, including 176 PTCs; among these 47 *BRAF^V600E^* mutated and 27 gene fusion positive (18 with *RET/PTC1*, and 9 with *RET/PTC3*) samples were available for testing.

For TCGA study (4), 227 *BRAF^V600E^* mutated and 28 gene fusion positive (21 with *RET/PTC1*, 4 with *RET/PTC3*, and 3 with *NTRK1*) PTC samples were evaluated.

**Reference List**

(1) Irizarry RA, Hobbs B, Collin F, Beazer-Barclay YD, Antonellis KJ, Scherf U, Speed TP. Exploration, normalization, and summaries of high density oligonucleotide array probe level data. Biostatistics 2003;4:249-64.

(2) Miller JA, Cai C, Langfelder P, Geschwind DH, Kurian SM, Salomon DR, Horvath S. Strategies for aggregating gene expression data: the collapseRows R function. BMC Bioinformatics 2011; 4:12:322.

(3) Ritchie ME, Phipson B, Wu D, Hu Y, Law CW, Shi W, Smyth GK. limma powers differential expression analyses for RNA-sequencing and microarray studies. Nucleic Acids Res 2015;43:e47.

(4) The Cancer Gene Atlas Research Network. Integrated genomic characterization of papillary thyroid carcinoma. Cell 2014;159:676-90.

(5) Robinson MD, Oshlack A. A scaling normalization method for differential expression analysis of RNA-seq data. Genome Biol 2010;11:R25.

(6) Robinson MD, McCarthy DJ, Smyth GK. edgeR: a Bioconductor package for differential expression analysis of digital gene expression data. Bioinformatics 2010;26:139-40.

(7) Anania MC, Cetti E, Lecis D, Todoerti K, Gulino A, Mauro G, Di Marco T., Cleris L, Pagliardini S, Manenti G, Belmonte B, Tripodo C, Neri A, Greco A. Targeting COPZ1 non-oncogene addiction counteracts the viability of thyroid tumor cells. Cancer Lett 2017;410:201-11.
